# Construction of a prognostic model for colorectal adenocarcinoma based on Zn transport-related genes identified by single-cell sequencing and weighted co-expression network analysis

**DOI:** 10.3389/fonc.2023.1207499

**Published:** 2023-09-26

**Authors:** Hua Chen, Ting Zhao, Jianing Fan, Zhiqiang Yu, Yiwen Ge, He Zhu, Pingping Dong, Fu Zhang, Liang Zhang, Xiangyang Xue, Xiaoming Lin

**Affiliations:** ^1^Department of Thoracic Surgery, The First Affiliated Hospital of Wenzhou Medical University, Wenzhou, Zhejiang, China; ^2^Department of Microbiology and Immunology, School of Basic Medical Science, Institute of Molecular Virology and Immunology, Institute of Tropical Medicine, Wenzhou Medical University, Wenzhou, Zhejiang, China; ^3^School of Second Clinical Medical, Wenzhou Medical University, Wenzhou, Zhejiang, China; ^4^Department of General Surgery, The Second Affiliated Hospital and Yuying Children’s Hospital of Wenzhou Medical University, Wenzhou, Zhejiang, China

**Keywords:** colon adenocarcinoma (COAD), Zn transport, colorectal cancer (CRC), prognostic, Immune infiltration, immune microenvironment Colon adenocarcinoma (COAD), immune microenvironment

## Abstract

**Background:**

Colorectal cancer (CRC) is one of the most prevalent malignancies and the third most lethal cancer globally. The most reported histological subtype of CRC is colon adenocarcinoma (COAD). The zinc transport pathway is critically involved in various tumors, and its anti-tumor effect may be through improving immune function. However, the Zn transport pathway in COAD has not been reported.

**Methods:**

The determination of Zn transport-related genes in COAD was carried out through single-cell analysis of the GSE 161277 obtained from the GEO dataset. Subsequently, a weighted co-expression network analysis of the TCGA cohort was performed. Then, the prognostic model was conducted utilizing univariate Cox regression and least absolute shrinkage and selection operator (LASSO) Cox regression analysis. Functional enrichment, immune microenvironment, and survival analyses were also carried out. Consensus clustering analysis was utilized to verify the validity of the prognostic model and explore the immune microenvironment. Ultimately, cell experiments, including CCK-8,transwell and scratch assays, were performed to identify the function of LRRC59 in COAD.

**Results:**

According to the Zn transport-related prognostic model, the individuals with COAD in TCGA and GEO databases were classified into high- and low-risk groups. The group with low risk had a comparatively more favorable prognosis. Two groups had significant variations in the immune infiltration, MHC, and the expression of genes related to the immune checkpoint. The cell experiments indicated that the proliferation, migration, and invasion of the HCT-116, DLD-1, and RKO cell lines were considerably increased after LRRC59 knockdown. It proved that LRRC59 was indeed a protective factor for COAD.

**Conclusion:**

A prognostic model for COAD was developed using zinc transport-related genes. This model can efficiently assess the immune microenvironment and prognosis of individuals with COAD. Subsequently, the function of LRRC59 in COAD was validated via cell experiments, highlighting its potential as a biomarker.

## Introduction

Colorectal cancer (CRC) is one of the most prevalent malignancies. It was reported in 2022 as the third most lethal type of cancer worldwide (1). By 2035, more than 2.5 million people would be suffering from the disease, with over 1.1 million predicted deaths (2, 3). With the development of surgical methods and drug therapy, novel treatment schemes for CRC have become increasingly mature (4). However, the long-term survival rate of advanced CRC continues to be poor (5). Approximately 20% of CRC patients are diagnosed at advanced stages with metastases due to the lack of early typical clinical symptoms and up to 50% of patients with initially localized disease are likely to develop metastases (3, 6). This is one of the main reasons for the low survival rates in patients with advanced CRC, while difficulties in early detection, delays in referral for treatment, and cultural beliefs and financial constraints are other causes (7–9). Metastasis of CRC predominantly occurs in the regional lymph nodes, lungs, liver, and peritoneum (3). Although the prognosis of patients with metastatic CRC has gotten better due to the introduction of liver and lung metastasis surgery and novel anti-tumor drugs, in most cases, there is still no cure (10). Molecular targeted therapy and immunotherapy, such as anti-VEGF monoclonal antibodies and immune checkpoint inhibitors, are being investigated for their value in CRC (11–13). However, certain CRC patients showed no improvement in overall survival after specific treatment, which could be associated with the low mutation load and the production of immunosuppressive factors (14, 15). As a result, researching new biomarkers and comprehending their role in the tumor microenvironment is critical in guiding treatment for CRC. Colon adenocarcinoma (COAD) is the most prevalent histological subtype of CRC, comprising over 90% of cases (16).

Zinc is an essential trace element within the human body that is vital for the maintenance of protein structure, enzyme activity, and gene regulation (17, 18). Zn transport-related proteins are involved in maintaining zinc homeostasis (19). The two most vital transporter families are ZIP (SLC39A) family, which promotes zinc influx into the cytoplasm, and ZnT (SLC30A) family, which promotes zinc efflux from the cytoplasm (20, 21). Zinc dyshomeostasis due to the dysfunction of Zn transport-related proteins has been shown to contribute to an elevated risk of developing several cancers, including prostate, breast, and pancreatic cancers (22–25). Zinc metabolism is closely related to anti-tumor, and its main mechanisms include DNA damage, DNA repair, oxidative stress, and immune function (26, 27). It is generally considered that its antioxidant and pro-apoptotic properties primarily manifest the protective effect of zinc on the occurrence of cancer by reducing oxidative stress and improving immune function (28, 29). In specific cancers, zinc deficiency can also lead to the loss of immature B cells and reduce antibody production (30). It is worth noting that several clinical studies have used plasma or serum to assess systemic zinc status as a biomarker of cancer patients and have found changes in zinc levels in serum and malignant tissues (31–33). The role of zinc transport-related proteins in breast cancer, prostate cancer, and pancreatic cancer has been extensively explored (22, 34–37), but little has been reported in CRC. Therefore, exploring the function of zinc transport-related proteins in CRC is of considerable significance.

This research acquired the data of individuals with COAD from the TCGA database and the GSE17538 dataset from the GEO databases. Through univariate Cox regression analysis and least absolute shrinkage and selection operator (LASSO) Cox regression analysis, a prognosis-predictive model of COAD patients according to Zn transport-related genes was developed. The individuals with COAD were classified into high- and low-risk groups as per their respective risk scores. Overall survival (OS) of individuals with COAD in both the training and external validation sets was remarkably elevated in the group with low risk than in the group with high risk. Additionally, this study explored the mutation profile and tumor immune microenvironment in both groups, and the correlation between both groups and MHC marker genes and immune checkpoints was analyzed. Lastly, the study verified the specific role of LRRC59 in the gene model through cellular functional experiments, including Cell Counting Kit-8 (CCK8) proliferation assay and Transwell invasion assay, demonstrating that LRRC59 has a potential to serve as a prognostic biomarker and a potential target that can help in the treatment of COAD. The findings of this research can be useful in diagnosing and treating COAD.

## Materials and methods

### Data acquisition and processing

RNAseq data and the clinical data of 514 individuals with COAD were retrieved from the TCGA (https://portal.gdc.cancer.gov/) as a training set. Clinical data for patients whose follow-up duration and recorded date of death were incomplete were excluded. The COAD dataset GSE17538 was downloaded from the GEO database as the external validation set. Patients without survival data were excluded from the cohort, and all the data were converted to log2 for the following analyses.

### The acquisition of genes linked to Zn transport

The genecards database (https://www.genecards.org/) was searched for 565 genes associated with Zn transport in December 2022.

### Downloading and processing of single-cell sequencing data

The single-cell sequencing dataset GSE161277 was retrieved from the GEO database containing four samples. Subsequently, data quality control was conducted. The cells with >50 genes among which<5% mitochondrial genes were retained. The number of highly variable genes was set at 1500. The integration of these four samples was performed using SCT correction. Next, the tSNE technique was applied to decrease the dimensionality of data, with the “DIMS” parameter set to 20. Cell clustering was conducted utilizing the “KNN” technique with a resolution of 1.0. Afterward, the R package “singleR” was employed to annotate the cells by different markers on the cellular surface. Lastly, the percentage of genes linked to Zn transport in all cells was acquired by importing Zn transport genes utilizing the “PercentageFeatureSet” function.

### Single-sample gene set enrichment analysis (ssGSEA)

To characterize the immune microenvironment of patients with COAD, based on the expression matrix of two risk groups, the ssGSEA analysis is employed to establish enrichment scores that indicate the level of enrichment of gene sets in each sample. In the current research, the scores associated with Zn transport in each sample of individuals with COAD were acquired by ssGSEA analysis using the R package “GSVA”.

### Weighted co-expression network analysis (WGCNA)

WGCNA is a systematical biology approach that characterizes patterns of gene correlation across various samples. This technique can be utilized to detect gene sets with high covariance and to select biological markers or therapeutic targets on the basis of the interconnectedness of the gene set and its link to phenotype. In the current research, the genes were sequenced from largest to smallest according to the median absolute deviation, and the top 5000 genes were selected for WGCNA utilizing the R package “WGCNA”. Then, the R package “pickSoftThreshold” was used to filter the power parameters in the range of 1 to 20 and select an appropriate soft threshold of 13. WGCNA was utilized to search for gene modules linked to Zn transport scores in COAD and obtain a list of effective genes related to Zn transport for subsequent analysis.

### Development of Zn transport-related prognosis-predictive model

Firstly, univariate Cox regression analysis was utilized to find the genes with the prognosis-predictive value that were related to Zn transport. Then, using the R package “glmnet”, LASSO Cox regression analysis aided in the selection of the genes related to Zn transport and develop a prognostic gene model. Finally, a multivariate Cox regression analysis was conducted to find independent predictive factors. The penalty parameter (λ) was quantified based on the minimum criteria, and 16 Zn transport-related genes were obtained along with their specific coefficients. The equation used to calculate the risk score is stated as:


$$\text{Risk socre }=\;\sum_{i=1}^{n}\left(Expression_{i}\times Coefficient_{i}\right)$$

### Validation of the prognosis-predictive model

The TCGA dataset was selected as the training set and the GSE17583 cohort in GEO was selected as the external validation set. Risk scores were computed for each sample in the training as well as the external validation sets using the risk score formula of the model. Individuals were classified into high- and low-risk groups as per their median risk score.

Then, with R package “survival” and “survminer”, Kaplan-Meier survival analysis was applied to draw Kaplan-Meier curves. Time-dependent ROC analysis was performed with the aid of the R package “timeROC,”, which generated ROC curves for OS over 1, 3, and 5 years.

### Correlation analysis of immune infiltration and genetic variations

The calculation outcomes for seven immune infiltration evaluation algorithms were downloaded from the TIMER2.0 database for all individuals in the TCGA database. Additionally, information on individuals with COAD was extracted. Then, the variations in immune cell infiltration between both risk groups were explored, and the heat map was applied to show the immune cells at various infiltration levels. The correlation box plot between two groups and marker genes of MHC was visualized utilizing the R package “ggplot2”. A total of 24 MHC molecules includes HLA-A, HLA-B, HLA-C, HLA-DMA, HLA-DMB, HLA-DOA, HLA-DOB, HLA-DPA1, HLA-DPB1, HLA-DPB2, HLA-DQA1, HLA-DQA2, HLA-DQB1, HLA-DQB2, HLA-DRA, HLA-DRB1, HLA-DRB5, HLA-DRB6, HLA-E, HLA-F, HLA-G, HLA-H, HLA-J, HLA-L.

Meanwhile, the genes linked to 35 immune checkpoints that were differentially expressed between both risk groups were illustrated as a box plot. Furthermore, the inter-group mutations in both risk groups were examined with the aid of the R package “maftools”, and we revealed the 15 genes with the most mutations. The tumor mutation load (TMB) of each sample was calculated by using the TMB function in the R package “maftools”.

### Establishment of a prognosis-predictive nomogram

A prognostic nomogram based on clinical features and risk scores was constructed by employing the R package “rms” and “survival”, which is uesd to predict the 1-, 3-, and 5-year OS of individuals with COAD. Then, the discrimination, calibration, and clinical effectiveness of the nomogram were illustrated through the calibration curve.

### Clinical correlation analysis

A heat map was displayed to investigate the variations in clinical features of COAD patients between both risk groups. Correlation analysis was conducted using the Chi-square test based on some significant clinical features.

### Consensus clustering

To determine distinct subtypes related to Zn transport, consensus clustering was conducted using k-means clustering. The R package “ConsensusClusterPlus” clustering algorithm was utilized to calculate the appropriate number of stable COAD clusters. We run 1000 iterations to ensure the accuracy and reliability of the final clustering, which was further verified by Kaplan-Meier analysis. A heat map was drawn to explore the expression of 16 genes linked to Zn transport after cluster analysis. Finally, the R package “GSEA” was employed to examine the immune infiltration levels between different clusters and drew a box plot for visualization. Sankey plots depicting the association between individuals in both risk groups and individuals with consensus clusters were generated using the R package “ggalluvial”.

### Functional enrichment analysis as per the gene model

Gene Ontology (GO) enrichment and Kyoto Encyclopedia of Genes and Genomes (KEGG) pathway analyses were performed utilizing the “clusterProfiler” package to investigate the specific function of the gene model and involved pathways. The analysis was based on 16 Zn transport-related genes, and the criteria used were |log2FC| > 1 and FDR< 0.05. Both plots were drawn with the aid of the R package “ggplot2”. Gene Set Enrichment Analysis (GSEA) was carried out to examine significant variations in the enrichment of the MSigDB cluster (c2.cp.kegg.symbols.gmt) gene set between both risk groups.

### Cell culture and transfection

The COAD cell lines, HCT-116,DLD-1, and RKO were supplied by the Cell Bank of the Chinese Academy of Sciences (Shanghai, China). HCT-116 and DLD-1 cell lines were placed in Roswell Park Memorial Institute 1640 Medium (RPMI 1640) (Gibco, Grand Island,NY) with the addition of 10% Fetal Bovine Serum(FBS;Sigma). RKO cell line was cultured in Minimum Essential Medium (MEM) (Gibco Grand Island,NY). HCT-116, DLD-1, and RKO cells were then transfected with the previously prepared LRRC59 small interfering RNAs for 24 hours utilizing the Lipofectamine2000 (Thermo Fisher Scientific, USA) following the provided guidelines. The LRRC59 siRNA was procured from RiboBio (Guangzhou,China).

### Cell viability

Cell viability was examined with the aid of the cell counting kit-8 (CCK-8) (Dojindo, Kumamoto, Japan). After seeding 4000 HCT-116, DLD-1, and RKO cells into 96-well plates and allowing them to adhere, siRNA (si-NC, si-LRRC59-1, si-LRRC59-2) transfection was performed. Following 24 hours, the cells were cultured with CCK-8 reagent at 37°C for 2 hours. The cell viability of HCT-116, DLD-1, and RKO cells was evaluated on days 1, 2, 3, and 4. Absorbance was measured at 450 nm utilizing a microplate reader. All statistical analyses were performed using the SPSS 17.0 software package (IBM, Chicago, IL) and were presented as the means ± SEM of three independent experiments. *P<0.05, **P<0.01, ***P<0.001.

### Quantitative real−time polymerase chain reaction (qRT-PCR)

qRT-PCR was conducted to assess the gene knockdown potential of the siRNAs. The 22-paired COAD tissues were collected from patients who underwent surgical resection for COAD at the Second Affiliated Hospital of Wenzhou Medical University (Wenzhou, China). The total RNAs was extracted using TRIzol Reagent and was reverse-transcribed with ReverTra Ace^®^qPCR RT Master Mix with gDNA Remover (TOYOBO, Japan). The qPCR reactions were conducted using Hieff^®^ Qpcr SYBR Green Master Mix (Yeasen Biotechnology (Shanghai)) in a 20µl reaction volume. Each reaction contained 10µl of 2×SYBR Green RT-PCR Master Mix, 0.4µl of each 10 µM forward and reverse primer, 1µl of cDNA sample, and nuclease-free water to make up the final volume to 20µl. The amplification process consisted of an initial denaturation step at 95°C for 5 minutes, followed by 40 cycles of denaturation at 95°C for 10 seconds and annealing at 60°C for 30 seconds. The relative expression of the gene was determined using the 2^-ΔCt method. The primers, the sequences of which are given in **Supplementary Table S1**, were provided by Sangon Biotech Co.,Ltd (Shanghai, China). All data were presented as the means ± SEM of three independent experiments. *P<0.05, **P<0.01, ***P<0.001.

### Immunohistochemical Staining of LRRC59

The protein expression level of LRRC59 (0.2725 mg/ml, HPA030829, Atlas Antibodies) in COAD and adjacent normal tissues was validated through immunohistochemical staining. The data was sourced from the Human Protein Atlas (HPA, https://www.proteinatlas.org/) database.

### Migration and invasion assays

To evaluate the migration and invasion of HCT-116, DLD-1, and RKO cells that had been transfected, twenty-four-well Transwell chambers with 8-um pore-size membranes were utilized. The transfected cell lines were administered into the upper chamber in serum-free medium. Thereafter, the medium containing 10% FBS was added to the lower chamber as a chemoattractant. The upper chamber was coated with or without Matrigel (BD Pharmingen, San Jose, CA) to assess the migration and invasion capacity, respectively. After incubating for 48 hours, the cells above the Matrigel layer were removed. The cells on the inserts were fixed using ice-cold methanol, stained with crystal violet, and counted under a microscope (Leica, UK) using four randomly selected fields per well. All data were presented as the means ± SEM of three independent experiments. *P<0.05, **P<0.01, ***P<0.001.

### Scratch would healing assays

To investigate the correlation between prognostic genes and tumor cell migration, we conducted scratch wound healing assays in both transfected and untransfected COAD cell lines (HCT-116, DLD-1, and RKO cells). Once the COAD cells reached 90-100% confluency in a 6-well culture plate, HCT-116 and DLD-1 cells were incubated in serum-free RPMI1640 (Gibco,Grand Island,NY) for 24 hours. On the other hand, RKO cells were cultured in MEM (Gibco, Grand Island,NY) for the same duration. After serum starvation, a straight line was created by scraping a row of COAD cells in each well using a sterile plastic straw. The cells were then washed twice with serum-free medium to eliminate any cellular debris. The scratch wounds were observed immediately (0 hours) and after 24 hours. Microscopic images were captured using an microscope (Olympus, Tokyo, Japan). To determine the extent of cell migration, the wound closure area was quantified by analyzing the images with Image J software. This experiment was repeated three times independently.

## Results

### Single cell sequencing data analysis

**Figure 1:**
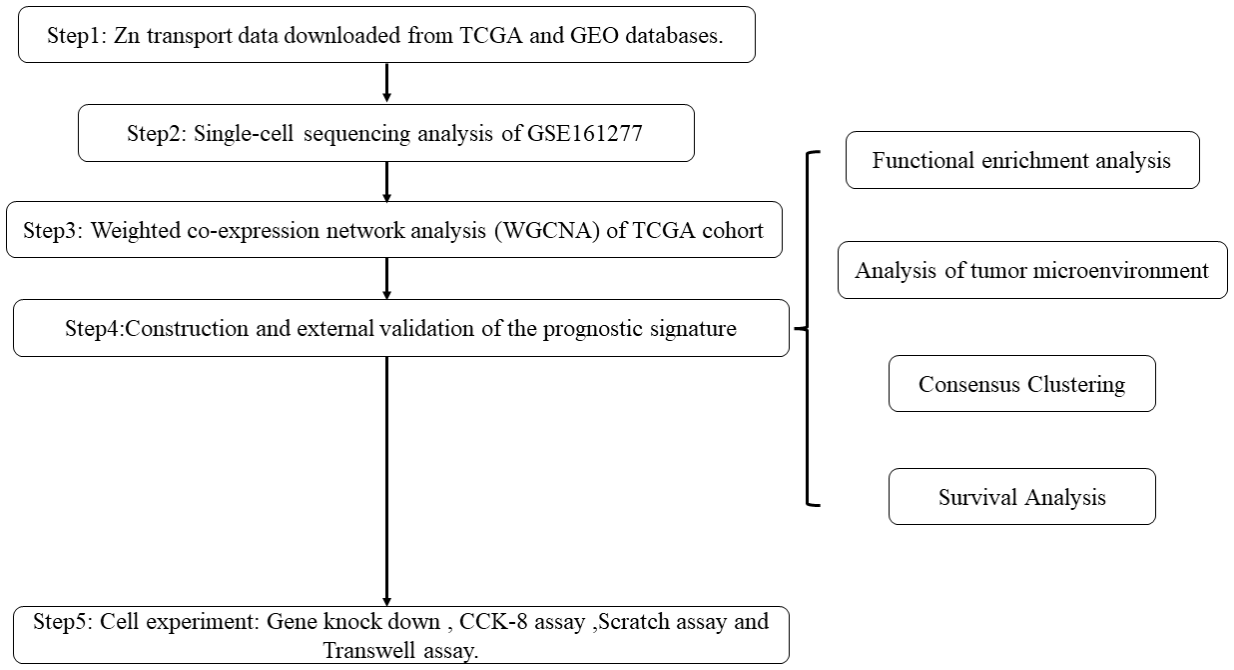
Flowchart of the study process.

The workflow of this research is displayed in **Figure 1**.

**Figure 2:**
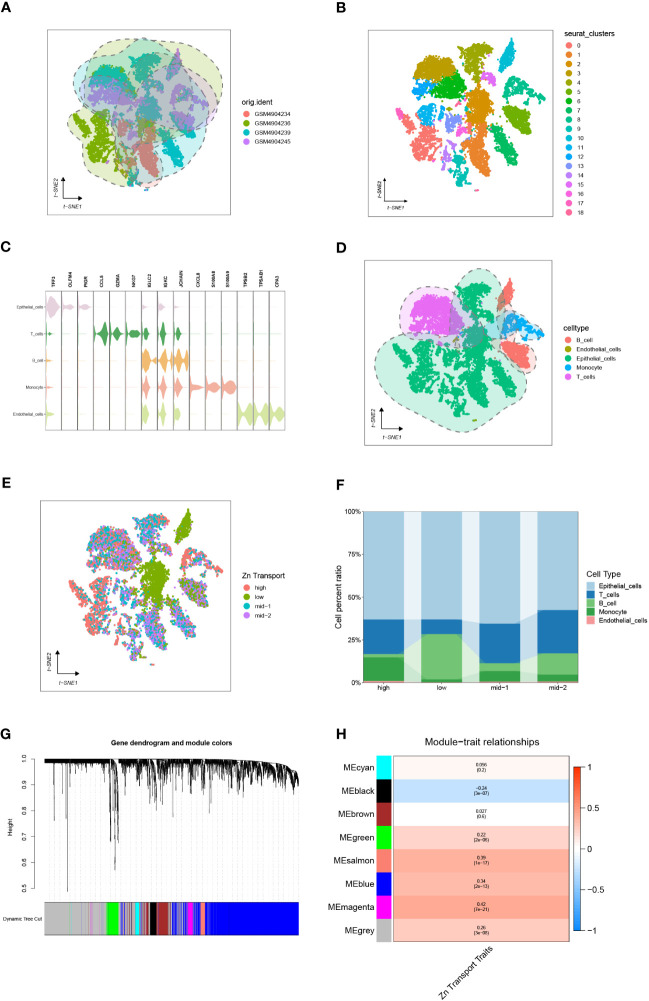
Single-cell sequencing analysis of GSE161277. **(A)** The integration effect of 4 samples. **(B)** Dimensionality reduction and cluster analysis. The cells from all 4 samples were clustered into 18 distinct clusters. **(C, D)** On the basis of surface marker genes of distinct cells, the cells are annotated as epithelial cells, T cells, B cells, monocytes, and endothelial cells. **(E)** The specific percentage of Zn transport-related genes in each cell. Cells were sorted into high-, low-, mid-1-, and mid-2-Zn transport cells. **(F)** The proportion of high-, low-, mid-1-, and mid-2-Zn transport cells in different subpopulations. **(G, H)** WGCNA showed that MEblack, MEgreen, MEsalmon, MEblue, and MEmagenta were closely associated with the score of Zn transport.

To integrate various samples, the initial step involved analyzing the COAD-related single-cell sequencing dataset GSE161277, as depicted in **Figure 2A**. The research findings indicated that the integration of the four samples was successful, and there were no prominent batch effects, making it appropriate for further analyses. Then, all the included cells were divided into 18 clusters utilizing k-Nearest Neighbor (KNN) clustering algorithm (**Figure 2B**, **Supplementary Figure S1**). Surface marker genes of distinct cells were examined, and their expression levels in various clusters were analyzed. This ultimately resulted in the identification of 5 cell types. It included epithelial cells, T cells, B cells, monocyte, and endothelial cells (**Figures 2C, D**).

Meanwhile, The “PercentageFeatureSet” function was employed to input 565 genes related to Zn transport. This was done to calculate the percentage of genes linked to Zn transport in all cells. The cells were segmented into low and high Zn transport-related cells according to their median ratio of Zn-transport-related genes.To make the variation obvious, mid-1 and mid-2 groups were set. The final results were displayed by the tSNE diagram and Column scale diagram(**Figures 2E, F**). 4189 genes were identified through variation analysis of the low and high groups.

### Weighted co-expression network analysis

WGCNA of 514 samples from the TCGA database acquired gene modules related to the Zn transport phenotype. By establishing the soft threshold at 13, setting the minimum number of module genes to 20, applying a deepSplit value of 2, and merging modules with a similarity score below 0.3, a total of 8 non-gray modules were generated (**Figure 2G**). The following consequences as shown in **Figure 2H** demonstrated that MEblack, MEgreen, MEsalmon, MEblue, and MEmagenta were closely associated with the score of Zn transport in the non-gray module. Genes from these five modules were chosen for further analysis.

### Development of prognostic model related to Zn transport

**Figure 3:**
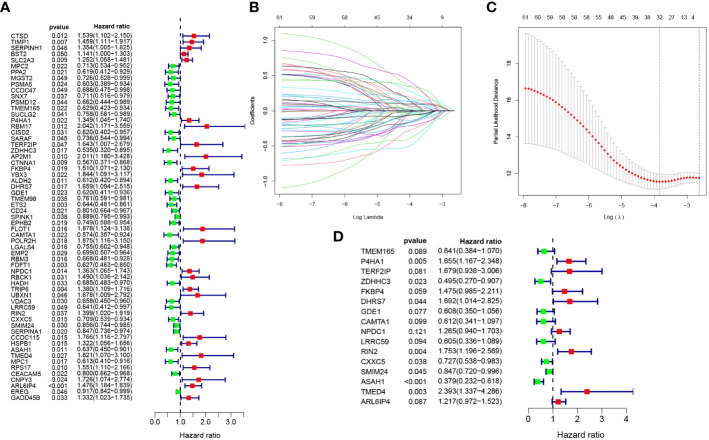
Development of Zn transport-related prognosis-predictive model. **(A)** Univariate Cox regression analysis to identify genes linked to prognosis. **(B, C)** A prognosis model was constructed based on 16 genes linked to Zn transport by LASSO regression analysis. **(D)** Multivariate Cox regression analysis of 16 Zn transport-related genes.

Firstly, 2821 genes were collected by intersecting the differentially expressed genes retrieved from single-cell sequencing data and Zn transport-related genes obtained by means of WGCNA. After matching the intersected genes obtained from both TCGA and GSE17538, 2583 genes were chosen for subsequent analyses. Additionally, by means of univariate Cox regression analysis on the TCGA cohort, 61 genes linked to the disease were identified (P< 0.05) (**Figure 3A**). Subsequently, LASSO Cox regression analysis was performed on 61 selected genes (**Figures 3B, C**). As per the optimum λ value, a prognostic gene model related to Zn transport was created utilizing 16 genes (TMEM165, P4HA1, TERF2IP, ZDHHC3, FKBP4, DHRS7, GDE1, CAMTA1, NPDC1, LRRC59, RIN2, CXXC5, SMIM24, ASAH1, TMED4, and ARL6IP4), whose coefficients are displayed in **Supplementary Table S2**. The risk score was derived as follows:

risk score = TMEM165 * -0.4443378 + P4HA1 * 0.503773089 + TERF2IP * 0.518042362 + ZDHHC3 * -0.702962146 + FKBP4 * 0.388985104 + DHRS7 * 0.526051475 + GDE1 * -0.496992354 + CAMTA1 * -0.491673477 + NPDC1 * 0.234995414 + LRRC59 * -0.502129112 + RIN2 * 0.561275588 + CXXC5 * -0.318620492 + SMIM24 * -0.165828979 + ASAH1 * -0.970779696 + TMED4 * 0.872693271 + ARL6IP4 * 0.196144485.

Additionally, multivariate Cox regression analysis was performed according to these 16 genes, and the corresponding results are illustrated in **Figure 3D**.

### Validation of the prognosis-predictive model

**Figure 4:**
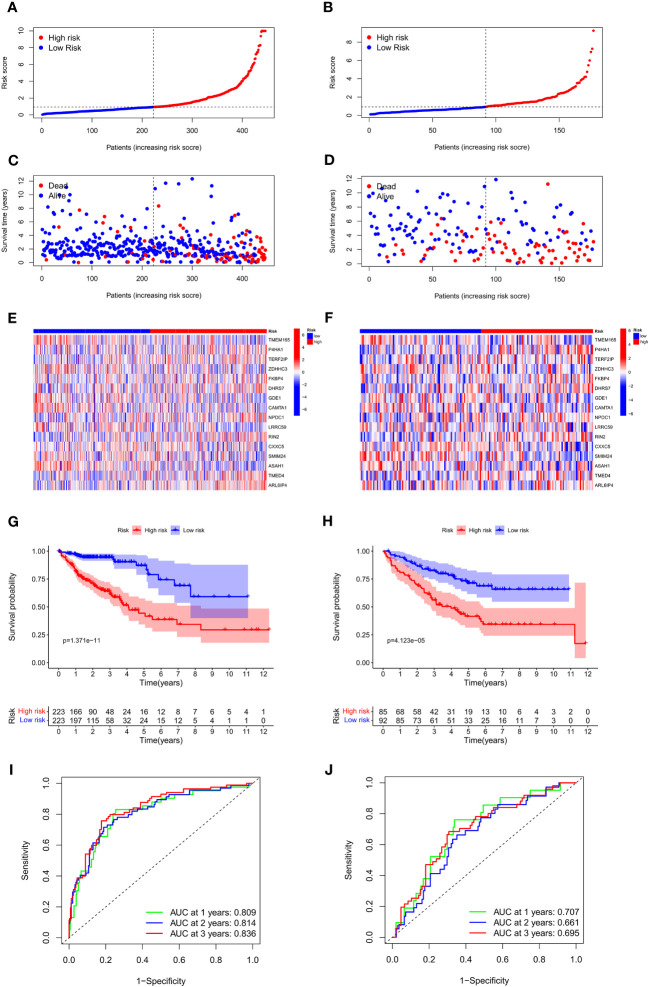
Association between the risk score and OS of individuals with COAD in the training and external validation set. **(A, B)** The distribution of risk scores in the training and validation set. **(C, D)** The survival status of patients in the training and validation set. **(E, F)** Heat map of 16 Zn transport-related genes expression in the training and validation set. **(G, H)** KM curves in the training and validation set. **(I, J)** Time-dependent ROC curves of the prognosis-predictive model to predict 1, 2, and 3 years in the training and validation set.

To assess and validate the performance of the prognosis-predictive model, the TCGA cohort was utilized as the training set, whereas the GSE17583 from the GEO database was utilized as the external validation set. Individuals were classified into low- and high-risk groups as per their median risk score (**Figures 4A, B**). Both in the training and validation sets, individuals with COAD in the low-risk group exhibited a favorable prognosis than those in the high-risk group (**Figures 4C, D**). The heat map was employed to display the expression of the 16 genes linked to Zn transport in both risk groups (**Figures 4E, F**). The Kaplan-Meier survival curves highlighted a considerably elevated probability of survival of individuals with COAD in the low-risk group compared to the individuals in the high-risk group, p = 1.371e-11 in the training set (**Figure 4G**) and p = 4.123e-05 in the validation set (**Figure 4H**).

Additionally, a receiver operating characteristic (ROC) curve analysis was conducted to evaluate the effectiveness of the prognosis-predictive model. The results of the training set in **Figure 4I** revealed that the area under the curve (AUC) values at 1, 2, and 3 years were 0.809, 0.814, and 0.836. As shown in **Figure 4J**, it was found that the respective AUC values at 1, 2, and 3 years were 0.707, 0.661, and 0.695. It is evident that the model had an efficient prognosis-predictive ability.

### Immune infiltration and genetic mutation analysis

**Figure 5:**
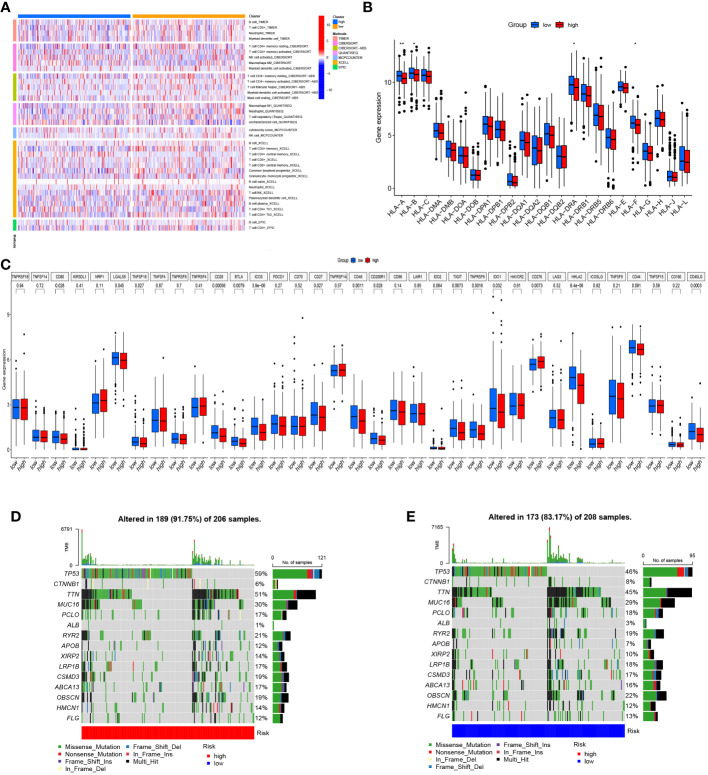
Immune infiltration and genetic mutation analysis. **(A)** Heat map of immune infiltration in both risk groups. **(B)** The link between both risk groups and MHC. **(C)** Differential expression of genes linked to the immune checkpoint in both risk groups. **(D, E)** Mutation status of both risk groups. *p< 0.05; **p< 0.01; ***p< 0.001.

Further analysis was performed to find the variations in immune infiltration levels between both risk groups, thus providing insights into immunotherapy for COAD patients. The findings highlighted that the individuals in the high-risk group exhibited more infiltration of macrophages and NK cells, while the individuals in the low-risk group had more infiltration of CD4 memory-resting T cells and CD4 memory-activated T cells (**Figure 5A**). **Figure 5B** illustrates the relationship between the two groups and marker genes of MHC, aiming to provide further insights into the significance of Zn transport-related genes in COAD treatment. It turns out that the expression levels of HLA-A, HLA-B, HLA-DRA, and HLA-F in the low-risk group were considerably higher than in the high-risk group. Next, the variations in the expressions of 35 genes related to immune checkpoint in both risk groups were explored. As highlighted in **Figure 5C**, most of these genes were confirmed to have remarkably elevated expression levels in the group with low risk, while CD276 was the only exception. The mutations in the 15 most mutated genes were analyzed in the two risk groups. It was confirmed that the mutation incidence of the individuals in the high-risk group was 91.75%, while that of the individuals in the low-risk group was slightly lower at 83.17%. Notably, the gene with the most significant mutation in both risk groups was TP53 (**Figures 5D, E**).

### Cell localization of modeling genes

**Figure 6:**
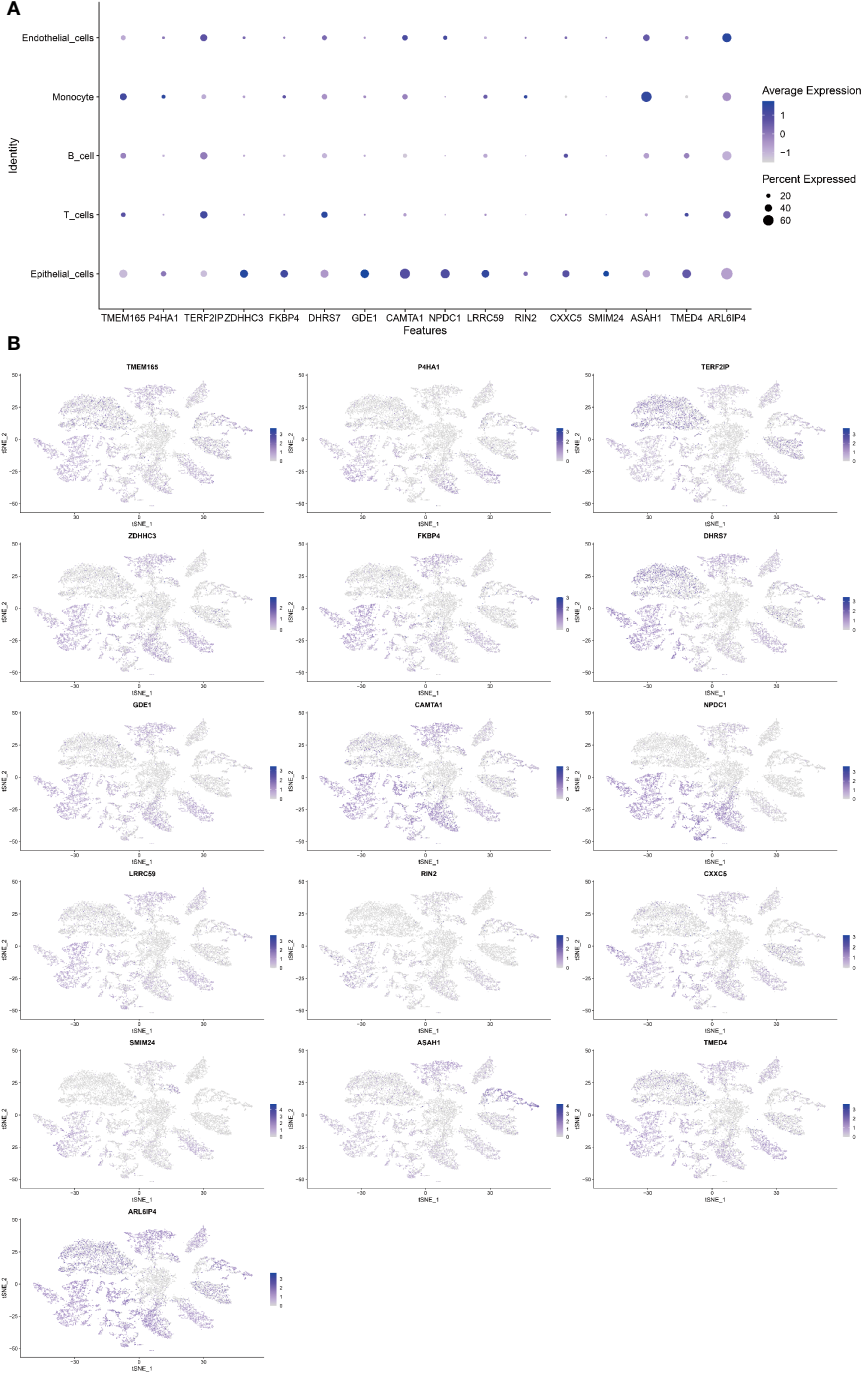
Single-cell sequencing analysis to investigate the cellular localization of 16 modeling genes. **(A)** The average expression of 16 model genes in 5 different cell types. **(B)** Specific localization of 16 model genes in 5 distinct cell types.

Single-cell sequencing analysis was utilized to investigate the expression levels of 16 modeling genes in distinct cell types, including endothelial cells, monocyte, B cells, T cells, and epithelial cells. As shown in **Figure 6A**, ARL6IP4 was primarily expressed in endothelial cells. TMEM165, P4HA1, RIN2, and ASAH1 were primarily expressed in monocyte. TERF2IP and DHRS7 were mainly expressed in T cells. ZDHHC3, FKBP4, GDE1, CAMTA1, NPDC1, LRRC59, CXXC5, SMIM24, and TMED4 were mainly expressed in epithelial cells. However, none of the model genes expressed primarily in B cells (**Figure 6B**).

### Development and validation of the prognosis-predictive nomogram

**Figure 7:**
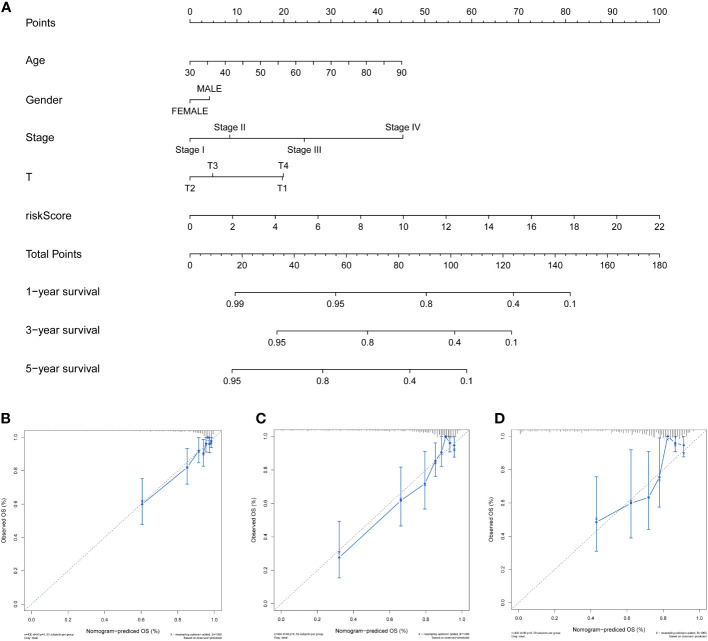
Development and validation of the prognosis-predictive nomogram. **(A)** Prognosis-predictive nomogram to predict OS probability of individuals with COAD at 1, 3, and 5 years. **(B–D)** Calibration curves of the nomogram to predict 1-, 3-, and 5-year OS probability in TCGA cohort.

To effectively and intuitively predict the prognosis of individuals with COAD, a prognostic nomogram was established utilizing the relevant prognostic factors (**Figure 7A**). The prognostic nomogram included variables such as gender, age, stage, T stage, and risk score. The total score was calculated by summing the scores related to each variable and was utilized to determine the survival probability of OS over 1, 3, and 5 years. To assess the performance of the constructed nomogram, 1-, 3-, and 5-year calibration curves were generated. A strong consistency between the observed and predicted values was observed (**Figures 7B-D**). Overall, the prognostic nomogram demonstrated an ideal predictive capacity for OS over 1, 3, and 5 years for COAD patients and therefore holds promise for clinical application.

### Clinical characteristics in the two risk groups

**Figure 8:**
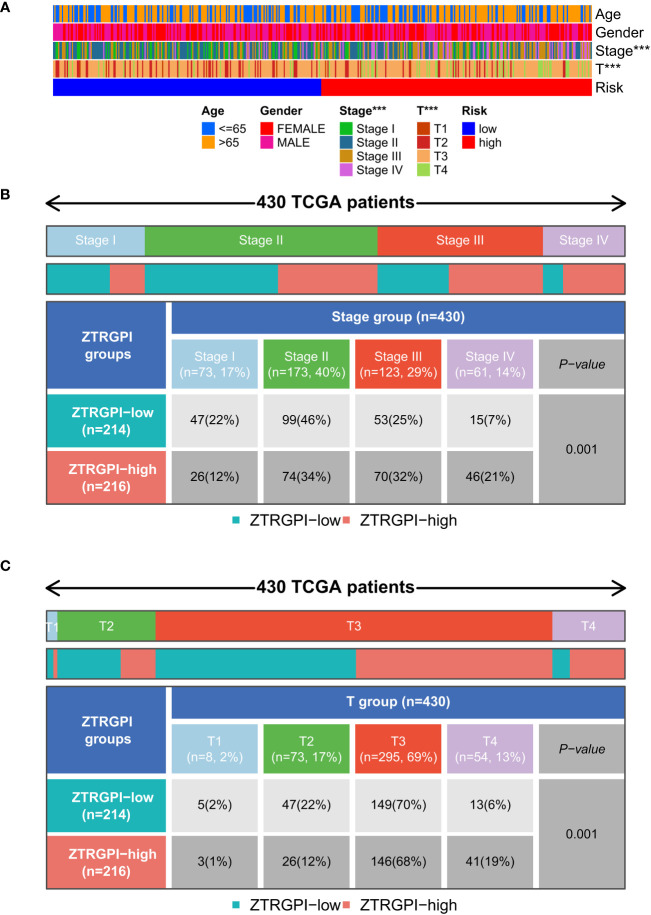
Clinical characteristic correlation analysis. **(A)** Clinical correlation analysis heat map. **(B)** Stage clinical correlation chart. **(C)** T-stage clinical correlation chart. ***p<0.001.

The link between clinical features, such as age, gender, stage, and T stage, and the risk signatures was analyzed to determine their distribution in both risk groups. The results were displayed in a heat map shown in **Figure 8A**. The results indicated more Stage I and Stage II patients in the ZTRGPI-low (Zn transport-related gene-based prognostic index) subgroup than in the ZTRGPI-high subgroup. Moreover, there were more Stage III and Stage IV patients in the ZTRGPI-high subgroup than in the ZTRGPI-low subgroup (**Figure 8B**). Similarly, this study revealed that the proportion of individuals with T1 and T3 stages was nearly equal between the two groups. Additionally, the ZTRGPI-high subgroup exhibited a greater number of T4 patients and a lower number of T2 patients compared to the ZTRGPI-low subgroup (**Figure 8C**).

### Determination of COAD classification based on 16 Zn transport-related genes

**Figure 9:**
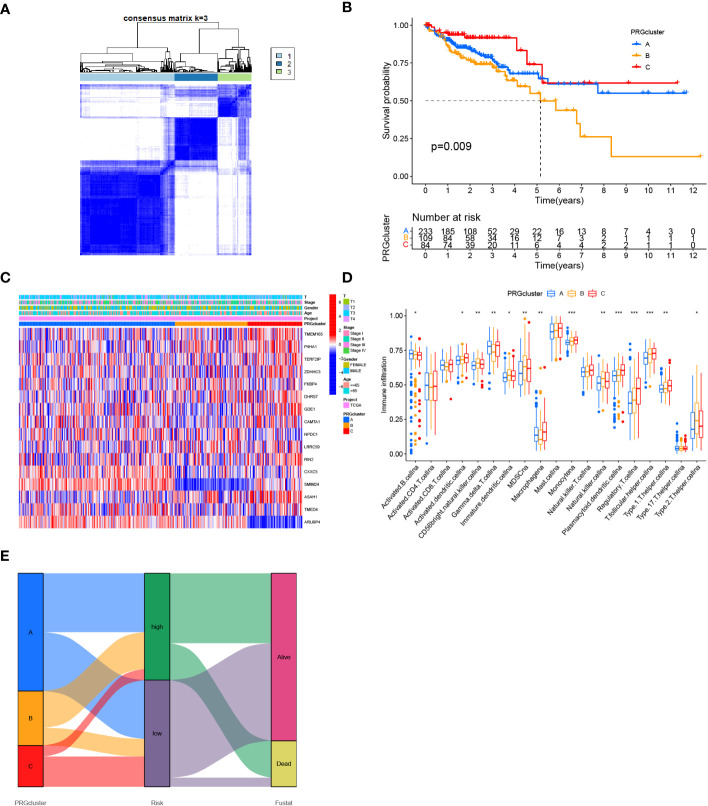
Determination of COAD classification based on Zn transport-related genes. **(A)** 424 individuals with COAD were classified into three clusters by the consensus clustering matrix (K = 3). **(B)** KM analysis of individuals with COAD in three clusters. **(C)** Heat map of the expression of 16 genes linked to Zn transport in classification and the link between clinical features and classification. **(D)** Box plot for ssGSEA analysis between three clusters. **(E)** Sankey diagram for three clusters and the two risk groups. *p< 0.05; **p< 0.01; ***p< 0.001.

Three clusters were identified utilizing consensus clustering methods in the TCGA cohort as per the expression of 16 genes related to Zn transport and patients’ clinical survival information. In addition, 224 samples were in cluster A, 114 in cluster B, and 86 in cluster C (**Figure 9A**). The survival analysis outcomes indicated that individuals with COAD in cluster C had a considerably worse OS time than those in Cluster B, and individuals in Cluster B had a remarkably worse OS time than those in cluster A (**Figure 9B**). A heatmap was created based on 16 Zn transport-related genes to investigate the variations across the three clusters. The heatmap demonstrated the expression profiles and clinical features of the 16 genes, including T stage, stage, gender, and age. The study revealed that the expression level of the majority of Zn transport-related genes, except for SMIM24 and ARL6IP4, exhibited a significant increase in cluster C (**Figure 9C**). In addition, the differences in immune infiltration across the three clusters were explored by means of ssGSEA. The findings highlighted that cluster A was only remarkably enriched in B cells, cluster B was remarkably enriched in CD56bright NK cells and NK cells, and cluster C was considerably enriched in monocyte, plasmacytoid dendritic cells, regulatory T cells, and T follicular helper cells (**Figure 9D**). According to the Sankey diagram, individuals who belonged to cluster A and low-risk groups exhibited a more favorable prognosis (**Figure 9E**).

### Functional enrichment analysis based on 16 Zn transport-related genes

**Figure 10:**
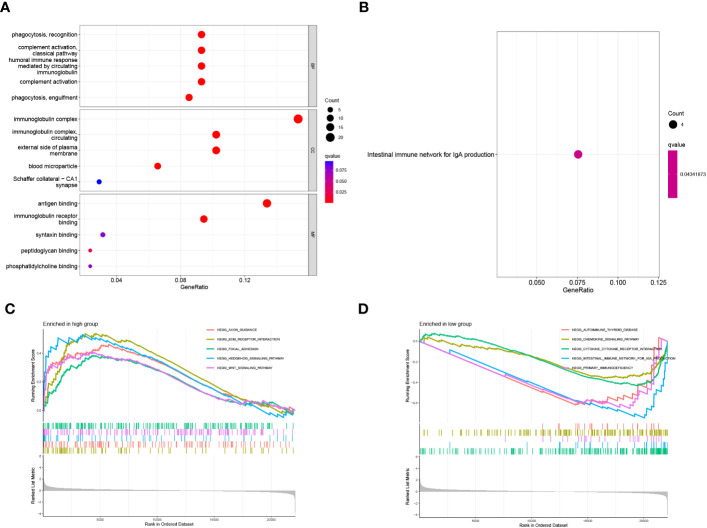
Functional enrichment analysis of 16 genes related to Zn transport. **(A, B)** The functions and pathways based on 16 genes linked to Zn transport by the analysis of GO and KEGG. **(C, D)** GSEA enrichment analysis in the two risk groups.

The possible involvement of 16 Zn transport-related genes among the three clusters was further determined by means of GO enrichment and KEGG pathway analyses. It is indicated that these Zn transport-related genes involve phagocytosis, recognition, complement activation, immunoglobulin complex, external side of cell membrane, antigen binding, and immunoglobulin receptor binding in GO enrichment analysis (**Figure 10A**). Furthermore, based on the KEGG analysis, it was determined that the findings were associated with the intestinal immune network for IgA secretion (**Figure 10B**). In order to get a detailed view of the underlying mechanisms of COAD and identify potential therapeutic targets, GSEA was conducted for making a comparison between both risk groups. The enriched signaling pathways identified in the group with high risk were related to ECM receptor interaction, hedgehog signaling pathway, focal adhesion, and Wnt signaling pathways (**Figure 10C**). The primary immunodeficiency, cytokine-cytokine receptor interaction, and intestinal immune network for IgA production signaling pathway were found to be enriched in the group with low risk (**Figure 10D**).

### Expression association and survival analysis of LRRC59

**Figure 11:**
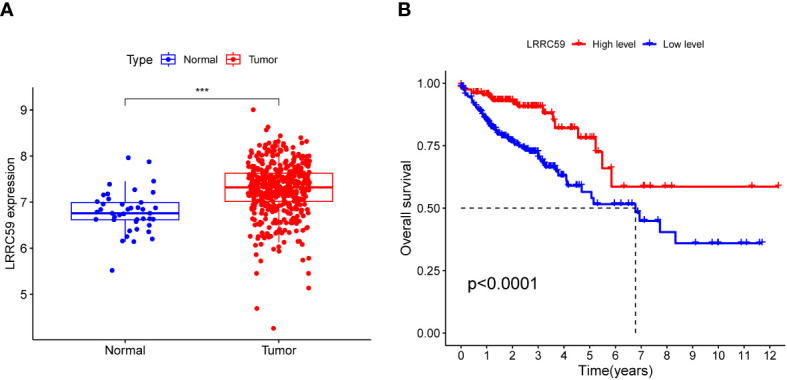
Expression association and survival analysis of LRRC59. **(A)** The expression level of LRRC59 was remarkably elevated in tumor tissues. **(B)** Individuals with COAD having lowered expression levels of LRRC59 had a considerably worse prognosis than individuals with elevated expression levels of LRRC59.

By analyzing the differential expression of LRRC59 in tumor and healthy tissues, it was found that the LRRC59 level was considerably elevated in tumor tissues (**Figure 11A**, ***p< 0.001). Further survival analysis highlighted that the group with elevated LRRC59 expression level exhibited a significantly higher survival rate compared to the group with lowered LRRC59 expression level (**Figure 11B**, p< 0.0001), suggesting that LRRC59 was a good protective factor.

### LRRC59 knockdown led to cell vitality of reduced COAD cell lines *In Vitro*

**Figure 12:**
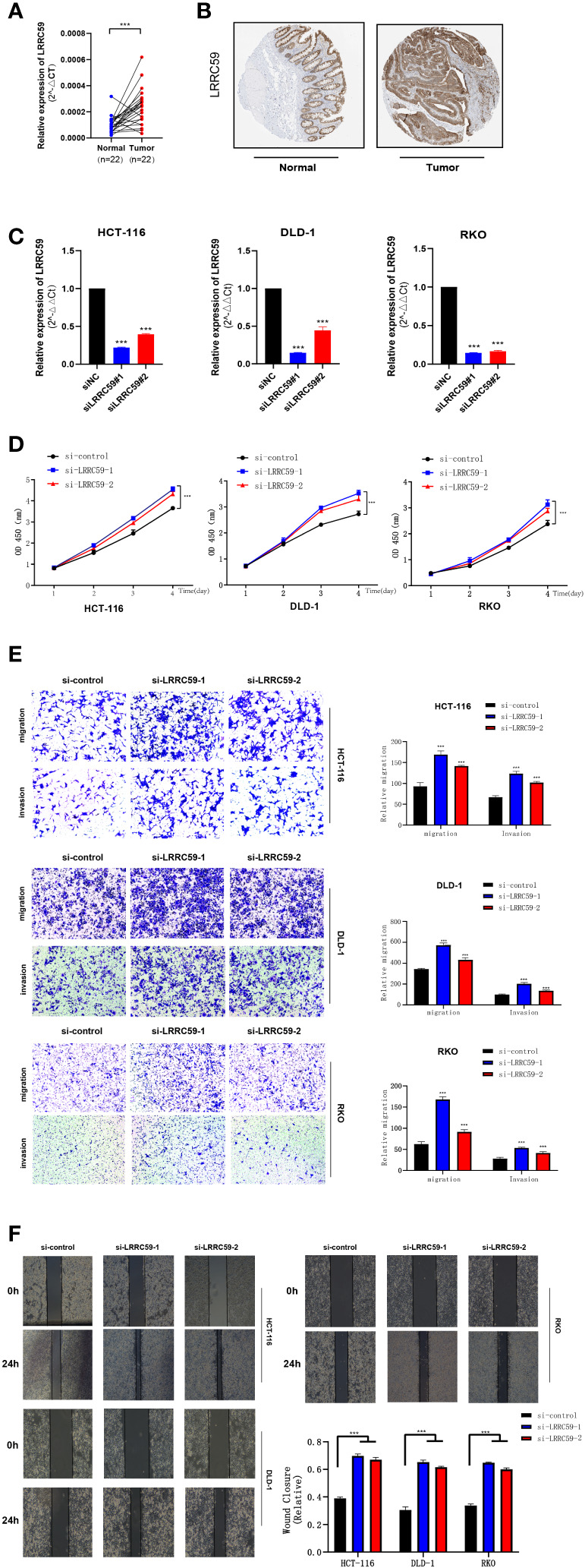
The expression of LRRC59 in patients with colorectal cancer and LRRC59 suppresses the proliferation, invasion, and migration of COAD cells. **(A, B)** The expression pattern of LRRC59 at the transcriptional and protein levels. **(C)** q RT-PCR to assess the level of LRRC59 mRNA 2 days after transfection. Both siRNA sequences could significantly decrease LRRC59 mRNA expression levels. **(D)** CCK8 assay. The cells indicated a considerable reduction in viability. **(E, F)** Transwell and Scratch assays. The migration and invasion ability of COADcells remarkably increased after LRRC59 knockdown. *p< 0.05; **p< 0.01; ***p< 0.001.

qPCR with 22 paired tumors and adjacent tissues was performed, suggesting that the mRNA expression of LRRC59 was significantly different from tumors and adjacent tissues (**Figure 12A**). Specific results revealed that all siRNA sequences caused a remarkable reduction in LRRC59 mRNA expression levels (***P<0.001). CCK8 assay indicated that cell viability increased significantly after LRRC59 gene knockdown, and si-LRRC59-1 and si-LRRC59-2 demonstrated effective knockdown potency, indicating their suitability for use in further *in vitro* experiments. It turns out that LRRC59 is critically involved in the survival of COAD cells **(** **Figure 12B**).

### Immunohistochemical staining of LRRC59

To validate the expression level of LRRC59, a prognostic marker gene, immunohistochemical staining results were obtained from the HPA database. The findings revealed that the intensity of LRRC59 immunohistochemical staining was higher in tumor cells compared to normal tissues, suggesting a significant upregulation of LRRC59 expression in tumor tissues compared to normal colon tissues.

### LRRC59 is critically involved in COAD cell lines migration and invasion *in vitro*

Subsequently, a Transwell assay was carried out, and the findings revealed a significant increase in the migration and invasion of HCT-116, DLD-1, and RKO cells following LRRC59 knockdown. It was found that the proportion of cells migrating across the pore plate was remarkably elevated after siRNA knockdown (**Figures 12C, D**, ***P<0.001). The scratch would healing experiments yielded comparable outcomes, indicating that wound healing rates were notably accelerated in cells exhibiting diminished LRRC59 gene expression (**Figures 12E, F**).

## Discussion

Colorectal cancer (CRC) is one of the deadliest malignancies and the third most prevalent contributor to cancer-related death globally, with individuals often presenting with metastatic disease (38). Less than 20 percent of patients diagnosed with metastatic CRC survive more than five years (39). The significance of immunotherapy in CRC treatment has been increasingly recognized by a growing body of research, among which immune checkpoint inhibition has indicated efficacy in the treatment of patients with metastatic CRC with mismatch-repair-deficient and microsatellite instability-high (dMMR-MSI-H) (40–42). Nonetheless, the exact mechanism of action of immune checkpoint inhibitors and other immunotherapies still needs further investigation. The overlapping metabolic reprogramming of tumor and immune cells is vital in activating the antitumor immune response (43, 44). Tumor metabolism is vital for sustaining signaling pathways in tumor onset and progression. It also has a wider impact on regulating the antitumor immune response by modulating the expression of immune molecules (45–47).

The protective effect of zinc transport-related genes on cancer is mainly manifested as reducing oxidative stress and enhancing immune system response (26). Zinc metabolism disorder may lead to zinc deficiency, thus causing thymus atrophy and lymphocytopenia, which impair cellular and antibody mediated immune response (30, 48).Several studies have also suggested that these genes can regulate metabolic fitness and enhance the antitumor effect through the metabolic reprogramming of immune cells (49, 50). In recent times, numerous models have been created by mining gene expression profiles and clinical characteristics of COAD, thus facilitating the investigation of the immune microenvironment of COAD (51, 52). Nevertheless, the diagnostic and predictive value of zinc transport-related genes in CRC is still poorly understood and valid evidence of zinc transport-related genes as targets for immunotherapy in CRC is lacking.

In our study, a prognosis-predictive risk model for individuals with COAD was constructed based on 16 genes linked to zinc transport, by means of univariate Cox regression and LASSO Cox regression analysis. This was achieved by a comprehensive assessment of COAD data obtained from TCGA and GEO databases. The calculation of the risk score enabled the classification of individuals with COAD into high- and low-risk groups. Considerably longer OS of individuals with COAD in the low-risk group was observed in both the training and external validation sets compared to that in the low-risk group. This is undoubtedly beneficial to the prognosis assessment of patients with COAD. In addition, the analysis of the immune infiltration level, immunotherapy response, and tumor mutation load highlighted variations in the immune microenvironment between both risk groups, which could potentially be beneficial for immunotherapy. The utilization of NMF consensus clustering methods enabled the identification of three clusters of COAD based on 16 zinc transport-related genes. Among these clusters, cluster A exhibited a better prognosis as compared to the other two clusters, subsequent studies can be further classified in Cluster A. Ultimately, our analysis showed that the expression of LRRC59 in COAD samples was significantly higher than that in normal samples, and according to the clinical data, the overall survival of patients in the group with high expression of LRRC59 was higher. The function of LRRC59 was validated by knocking down its expression. The findings indicated a significant improvement in the activity, proliferation, and invasion ability of COAD, suggesting that LRRC59 may serve as an early prognostic biomarker and a therapeutic target in COAD.

The significance of the zinc transport pathway in tumor development as well as the study of the immune microenvironment is becoming progressively more evident (22). An increasing number of evidence suggests that improving zinc transport metabolism and regulating the zinc transport signaling pathway may become a new approach for tumor therapy (53–55). The significance and action pathway of zinc transport in several types of tumors have been initially elucidated (56, 57). Certain study revealed that the upregulation of Ras-responsive element binding protein 1 (RREB1) led to the downregulation of zinc transporter 1 (ZIP1) and influenced zinc reduction in prostate cancer (58). Another study found that the downregulation of zinc transporter 3 (ZIP3) and RREB1 coincided with zinc loss during the early progression of pancreatic cancer and may help malignant cells eliminate the cytotoxic effects of zinc (59). Moreover, there was evidence demonstrated that the interaction of potassium channel tetramerization domain-containing 9 (KCTD9) and zinc transporter 9 (ZnT9) attenuated the expression of the β-catenin target gene and the inhibition of the Wnt signaling pathway. Finally, CRC cell proliferation and migration were inhibited (60). However, studies related to COAD and zinc transport are still lacking. Our study is the first to provide the prognostic biomarkers of zinc transport-related genes in COAD and to explore the immune microenvironment. These outcomes hold significant value for prognosis prediction and treatment of COAD individuals, and also provide help for further exploration of specific mechanisms of zinc transport regulation of tumor metabolic reprogramming.

CRC is characterized by high heterogeneity at the genetic and molecular levels, which can greatly affect the effectiveness of immunotherapy (42). At present, more and more reports have explored immune cell infiltration in the CRC microenvironment, patients with a better prognosis for CRC had a higher proportion of infiltrating CD8 and CD4 T cells, especially Th1 cells (61–64). This is consistent with the analysis results in this paper that patients in the low-risk group had a higher proportion of CD4 memory-resting T cells and CD4 memory-activated T cells. Different subtypes of CRC present a heterogeneous immune pattern (65). Most individuals with CRC have MSS tumors and poor immune cell infiltration (66). However, a small percentage of individuals with MSI-type tumors exhibit tumors that are enriched with immune cells, thereby activating the antitumor immune response (66). Immunotherapy is now gaining more and more attention in antitumor progression, with both immune checkpoint targeting and immunomodulatory monoclonal antibodies (mAbs) being developed (67, 68). Hence, it is crucial to comprehend the immune microenvironment of COAD. Based on zinc transport-related genes, this study indicated considerable variations in levels of immune cell infiltration between both risk groups, with more infiltration of macrophages and NK cells in the high-risk group and more infiltration of CD4 memory-resting T cells and CD4 memory activated T cells in the low-risk group. What’s more, The low-risk group was linked to elevated expression levels of immune checkpoint-related genes. Thus, the benefit of immunotherapy via immune checkpoint was higher in the low-risk group.

Leucine-rich repeat-containing protein 59 (LRRC59) is a ribosome-binding protein that also can interact with fibroblast growth factor (69–71). Research has demonstrated a correlation between alterations in LRRC59 expression and the metastatic and invasive potential of breast cancer cell lines (72). Furthermore, It is reported that a strong correlation between elevated LRRC59 expression levels and the survival rate of individuals with lung adenocarcinoma (LUAD) and demonstrated that reducing LRRC59 expression could considerably suppress the migration and invasive capabilities of LUAD cells (73). Nevertheless, the involvement of LRRC59 in COAD is yet to be studied in further detail. The investigation revealed, for the first time, that LRRC59 is a crucial protective factor in the modeled gene list, and subsequent survival analysis indicated that LRRC59 might serve as an independent prognostic factor. Finally, cell function experiments demonstrated that the knockdown of LRRC59 in COAD cell lines substantially increased cancer cell proliferation and invasion, which was contrary to previous studies of LRRC59 in LUAD, suggesting that LRRC59 plays a different mechanism of action in LUAD and COAD. These results further support the notion that LRRC59 has the potential to serve as a prognosis-predictive biological marker, thus aiding in the treatment of COAD.

The GSE161277 dataset has initially revealed the heterogeneity of abnormal epithelial cells and the complexity of the tumor microenvironment (74). It is indicated that the GSE161277 dataset has been utilized for single-cell analysis to examine the immune typing and tumor microenvironment of rectal cancer (75). This study first classified the COAD cells into two risk groups based on distinct zinc transport states through single-cell analysis of GSE161277, which provided a basis for the subsequent research of zinc transport heterogeneity in COAD and the development of the prognosis-predictive model. A prognosis-predictive model based on 16 genes related to zinc transport was developed and subsequently validated with the GSE17538 dataset. The findings indicated that the model can better assess the 1, 3, and 5 years OS of COAD patients. According to the possible relationship between zinc transport and tumor microenvironment, the heterogeneity of the COAD microenvironment in both risk groups was explored. The findings indicated that there were considerable variations in immune infiltration between the two groups. In subsequent immune checkpoint correlation analysis, it was observed that most genes related to immune checkpoints exhibited high levels of expression in the low-risk group, but CD276 displayed the opposite pattern. All of these provide references for the study of COAD immunotherapy and subsequent antitumor immune mechanism.

According to the current literature, the present research has highlighted the development of the first prognosis-predictive model based on genes linked to zinc transport by means of single-cell cluster analysis. This model serves as a valuable resource for the investigation of zinc transport in COAD and aids in the development of treatment strategies for individuals with COAD. At the same time, this study obtained a new biomarker of COAD and explored the association between tumor immune microenvironment and zinc transport.

## Conclusion

A prognosis-predictive model for COAD was developed based on genes related to zinc transport. This model has demonstrated the ability to effectively assess the prognosis and immune microenvironment of individuals with COAD. Subsequently, the function of LRRC59 in COAD was verified via cell experiments, thus highlighting its potential as a biomarker.

## Data availability statement

The datasets presented in this study can be found in online repositories. The names of the repository/repositories and accession number(s) can be found in the article/**Supplementary Material**.

## Ethics Statement

The studies involving human participants were reviewed and approved by Medical Ethics Committee of the Second Affiliated Hospital of Wenzhou Medical University. The patients/participants provided their written informed consent to participate in this study.

## Author contributions

XL, XX, and LZ desigened and directed all the research. HC, TZ performed the RNA-seq analyses, single cell analyses and cell phenotype experiment. JF, YG drafted the manuscript. The RNA extraction,reverse transcription, and qPCR were performed by ZY and HZ. PD and FZ participated in the revision of the paper. All authors reviewed the manuscript. HC, TZ, and JF contributed equally to this work. All authors contributed to the article and submitted and approved the submitted section.
