# Supplementary figures and images for: Construction of a prognostic model for colorectal adenocarcinoma based on Zn transport-related genes identified by single-cell sequencing and weighted co-expression network analysis
Source: Front Oncol. 2023 Sep 26;13:1207499. doi: 10.3389/fonc.2023.1207499 (PMC10565862; doi:10.3389/fonc.2023.1207499)

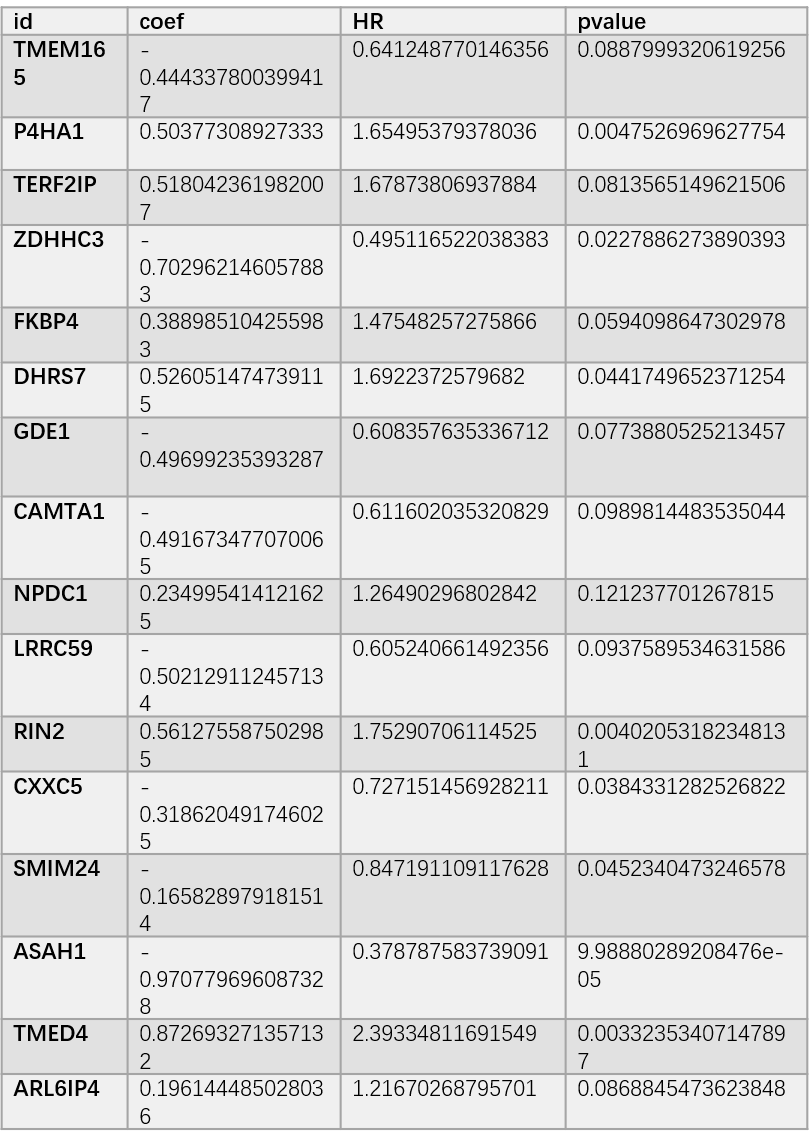

Supplement: Supplementary file 2 [file Image_1.png]

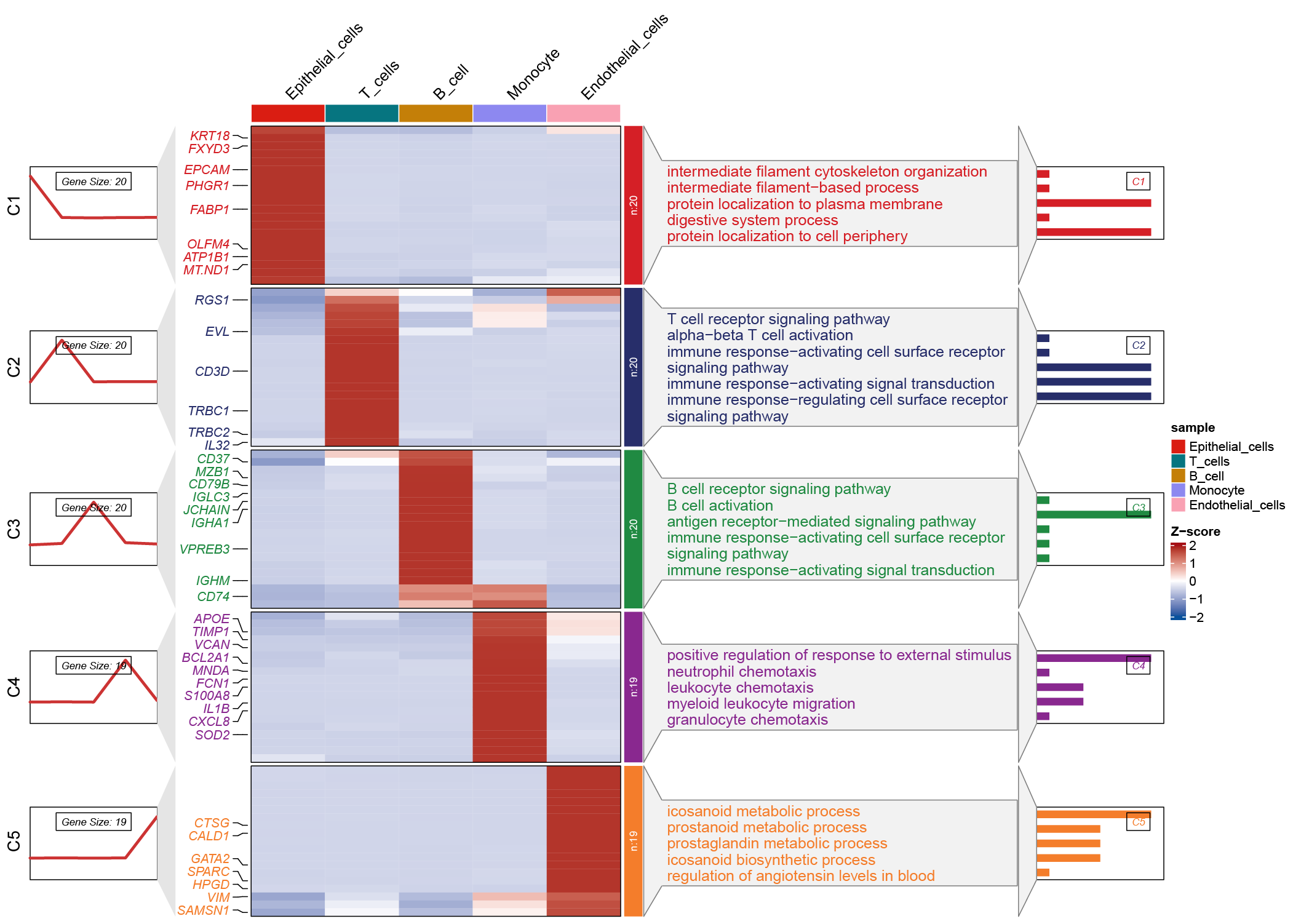

Supplement: Supplementary file 3 [file Image_2.tif]

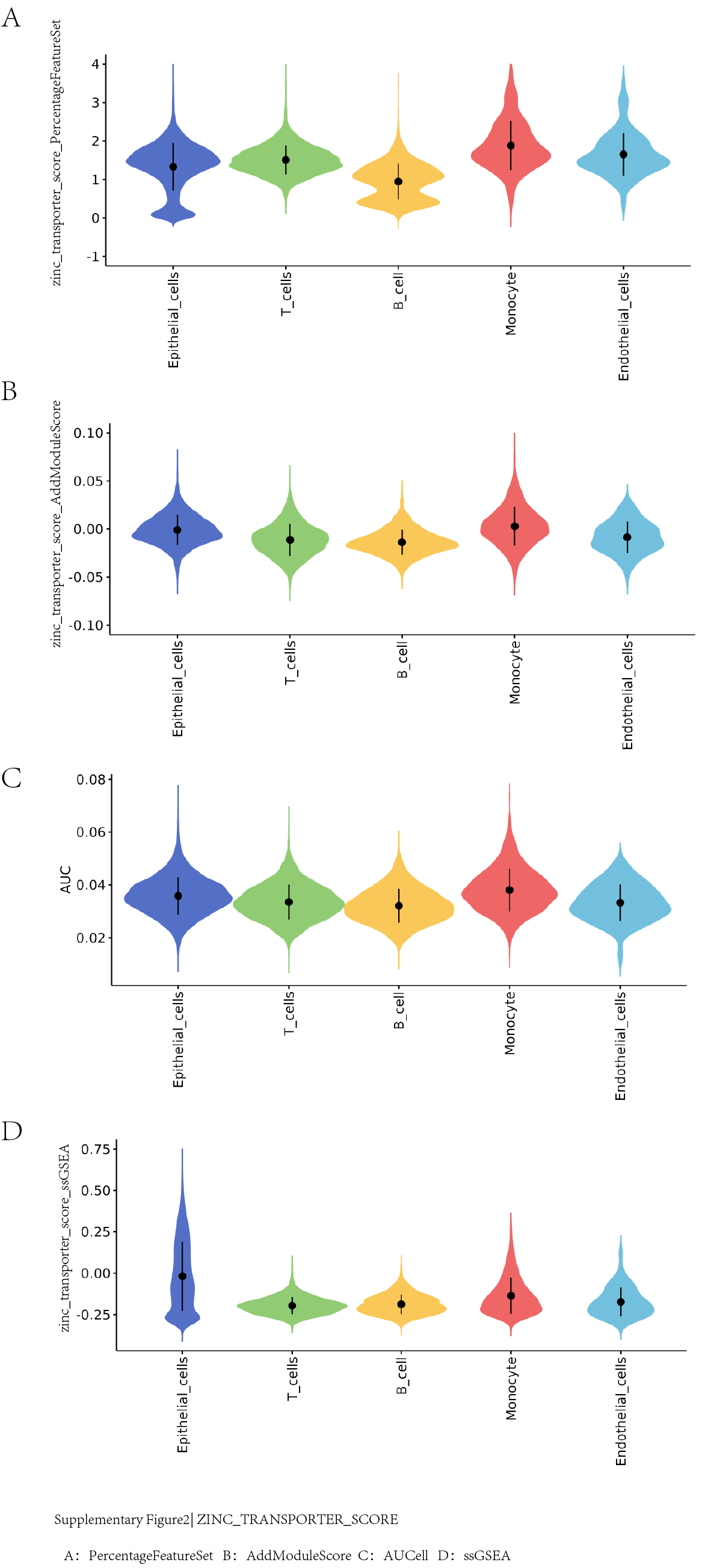

Supplement: Supplementary file 4 [file Image_3.tif]
